# Aqueous ionic effect on electrochemical breakdown of Si-dielectric–electrolyte interface

**DOI:** 10.1038/s41598-020-73880-w

**Published:** 2020-10-08

**Authors:** Jeongse Yun, Jae Gyeong Lee, Kyungbae Oh, Kisuk Kang, Taek Dong Chung

**Affiliations:** 1 Department of Chemistry, Seoul National University, Seoul, 08826 Republic of Korea; 2 Department of Materials Science and Engineering, Research Institute for Advanced Materials (RIAM), Seoul National University, 1 Gwanak-ro, Gwanak-gu, Seoul, 08826 Republic of Korea; 3 Institute of Engineering Research, College of Engineering, Seoul National University, 1 Gwanak-ro, Gwanak-gu, Seoul, 08826 Republic of Korea

**Keywords:** Chemistry, Electrochemistry, Inorganic chemistry, Materials chemistry, Physical chemistry

## Abstract

The breakdown of thin dielectric films (SiO_2_, Si_3_N_4_, HfO_2_) immersed in aqueous electrolyte was investigated. The current and the kinetics of dielectric breakdown caused by large cathodic electric field applied across the dielectric layer reveal the electrochemical nature of dielectric materials. Electrolytes play a huge role in the established dielectric-electrolyte interface with respect to the overall electrical behavior of the system. Although aqueous cations are considered as spectator ions in most electrochemical systems, in dielectric interfaces the current–potential characteristics depend on the type of cation. Computer simulation based on density functional theory and molecular dynamics showed cations affect the dielectric strength. The responses of various dielectric films to solution components provide invaluable information for dielectric-incorporated electrochemical systems.

## Introduction

The electrical conduction of inorganic insulating materials has been of keen and steady interest because it is critical for stability and reliability of electronic devices. Recently, the importance of insulators is rekindled due to its new found applications to batteries^1–3^, catalysts^4–6^, fundamental electrochemistry^7^, and iontronic devices^8^. The insulating thin film is more than an electrical and mechanical barrier protecting the underlying conductive material^9^. In electrochemical systems, it gives rise to certain characteristics that govern overall behavior, the reason for which is yet explored. For instance, unusual electrochemical phenomena is observed at SiO_2_^4,10,11^, an extensively studied insulator.

Concerning the role of insulator as a material where the mass transport and electrochemical reactions of various species could take place, research hitherto has been focusing on mass transport and the redox process of cation species in the dielectric layer. Most of the literature concern hydrogen species and alkali metal ions such as sodium ion and potassium ion. There are many reports supporting that metal cations possibly transfer from solution to the insulating films such as Si_3_N_4_^12,13^, HfO_2_^14^ and Al_2_O_3_^2,3,15,16^. Particularly, proton involvement under the electrochemical reaction condition has been reported because they are expected to form quasi-stable states in the molecular chain of insulating materials. For example, SiO_2_ film becomes conductive after extensive contact with acidic solution for several hours^10^, but electric current hardly flows at as-prepared-dry SiO_2_ film^17^. Lee et al. showed electrochemical reactions mediated by hydrogen species within SiO_2_ thin films^4,18^.

Dielectric breakdown (DB) is the loss of electrical insulation under applied electric field. Current leaks through the dielectric layer when applied electric field and cleaves chemical bonds which lead to local disruption and loss of insulating property^19^. Its kinetics reflects the physical and chemical environment around the dielectric material. Therefore, DB can be indicative of the process of impregnation by cationic species. Briggs et al*.* studied how the pH of electrolyte affects the DB of Si_3_N_4_^12^. In addition to protons, various metal ions such as sodium and potassium ions were investigated with detailed physical and chemical models to characterize device failure^20–23^. It is believed that metal ions, which were impregnated during the fabrication process and the device operation, can move within the dielectric oxides. Yet how the metal ions travel within the dielectric layer and modify its properties is controversial. Impregnation of ions into the insulators mostly involves harsh conditions so that the intrinsic nature of insulator undergo severe changes far from its pristine state^24–30^, resulting in the inconsistency of experimental results. Therefore, the study on this type of system require a less invasive method that can minimally modify the property of as-deposited insulator film.

In this work, we investigated DB of commonly used dielectric films, SiO_2_, Si_3_N_4_ and HfO_2_ on a highly doped Si electrode, to which negative potential bias is applied. The electrochemical behavior and DB kinetics allows monitoring of the effect of metal ions on the electrolyte–oxide–semiconductor (EOS) system.

## Results and discussion

**Figure 1:**
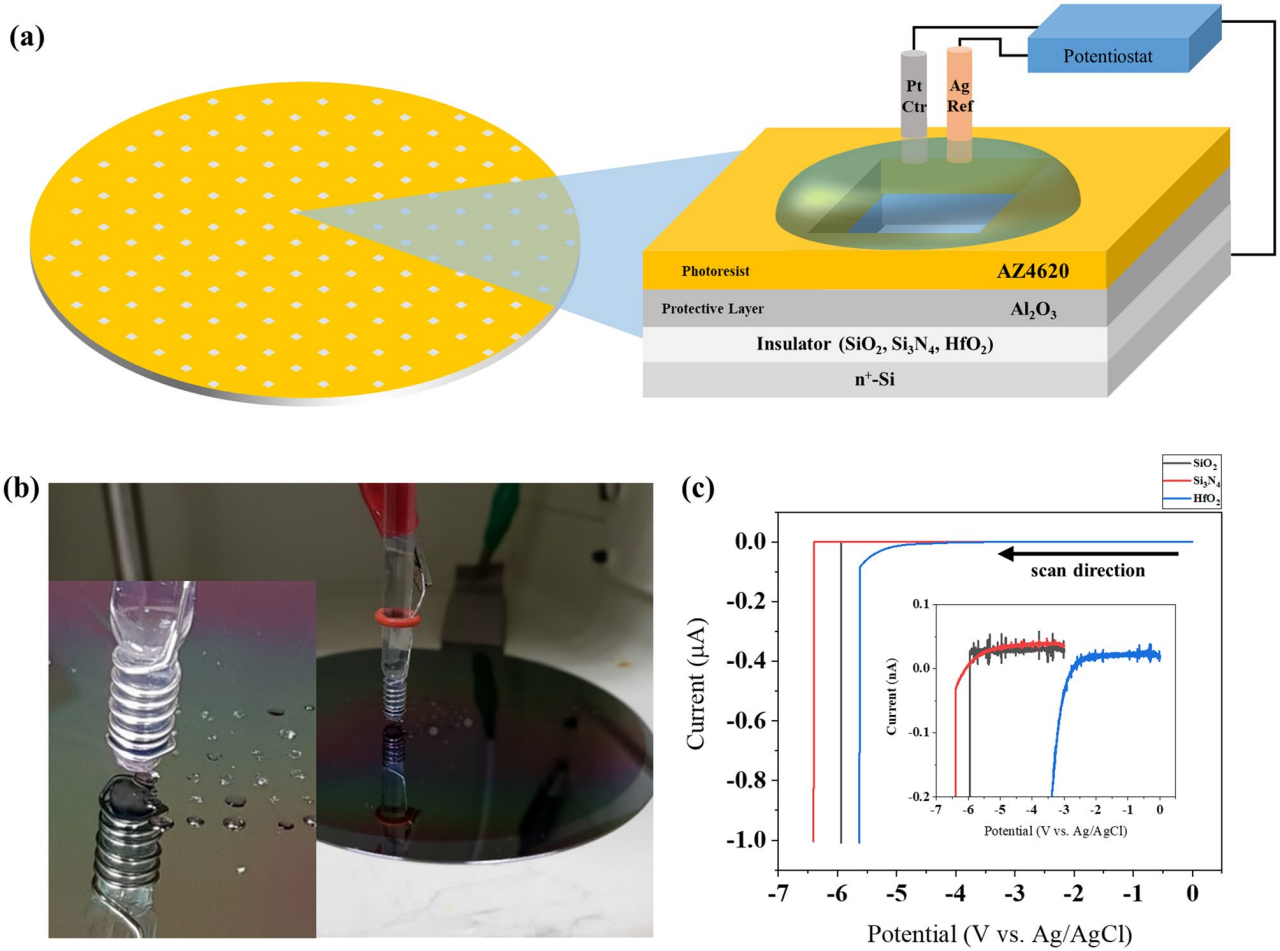
(**a**) Schematic diagram of electrochemical setup used in this study. Alumina and photoresist define the exposed area of insulators at 50** × **50 μm^2^. Whole Silicon wafer serves as a working electrode to minimize unwanted physical damage during dicing or manual handling. Ctr is Pt wire counter electrode and Ref is Ag/AgCl (3 M KCl) reference electrode with a double junction filled with 1 M KNO_3_. (**b**) Image of the electrochemical cell. (**c**) Typical current–potential curve of Si-insulator (10 nm)-aqueous electrolyte system.

Figure 1a,b show the experimental setup of the three-electrode configuration connected to a potentiostat. Typical voltammograms of SiO_2_, Si_3_N_4_ and HfO_2_ deposited on Si obtained in 0.5 M KCl are presented in Fig. 1c. The current surge indicates dielectric breakdown (DB) of the thin insulating film. Indicated on the *i*–V curves of SiO_2_, Si_3_N_4_ and HfO_2_ is the dielectric breakdown potential, *V*_db_. Current starts to increase before *V*_db_ for Si_3_N_4_ and HfO_2_, but for SiO_2_ only a small charging current flows. Band gap for Si_3_N_4_ and HfO_2_ is 5.3 eV^31^ and 5.7 eV^32^, respectively, which are much smaller than 9 eV for SiO_2_. This explains the current flow through the film under smaller bias at Si_3_N_4_ and HfO_2_. Before the breakdown, the electric field where leakage current begins to flow is about 6 MV/cm, 2 MV/cm, and 1 MV/cm for SiO_2_, Si_3_N_4_, and HfO_2_, respectively^33,34^. Inset of Fig. 1c shows a similar trend, therefore the electrolyte is not responsible for the exponential leakage current that appears before DB. Leakage current before breakdown flows through HfO_2_ unlike SiO_2_ and Si_3_N_4_, which is supposedly related to defects such as oxygen vacancies^35^. The breakdown voltages of HfO_2_ were taken where the current surges in Fig. 1c.

**Figure 2:**
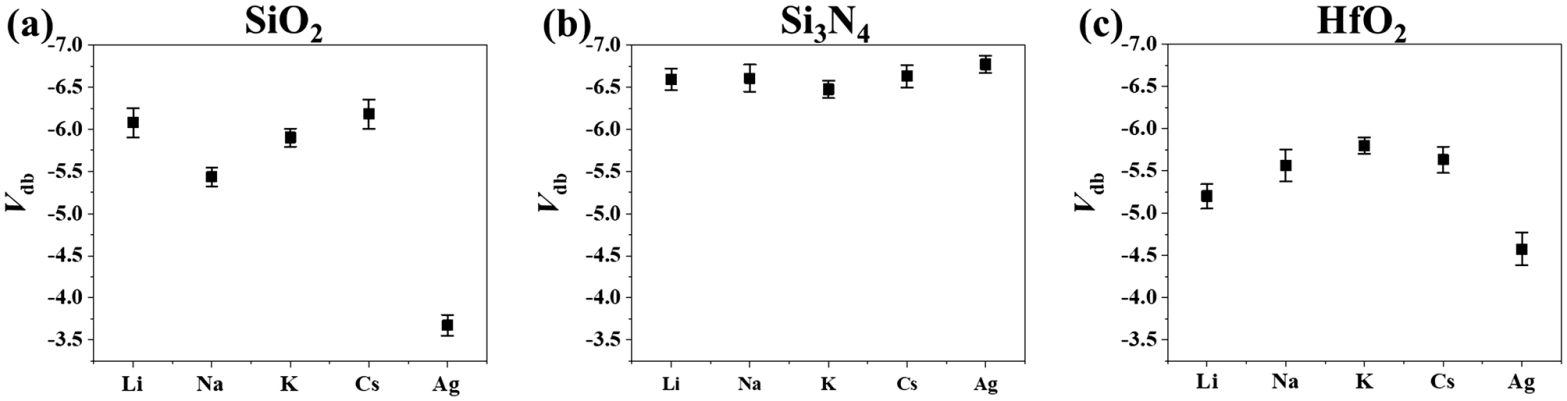
Potential at which dielectric breakdown occurs (*V*_db_) depending on metal cations in aqueous electrolyte. (**a**) Si–SiO_2_ (10 nm)-electrolyte, (**b**) Si–Si_3_N_4_ (10 nm)-electrolyte, (**c**) Si–HfO_2_ (10 nm)-electrolyte.

**Figure 3:**
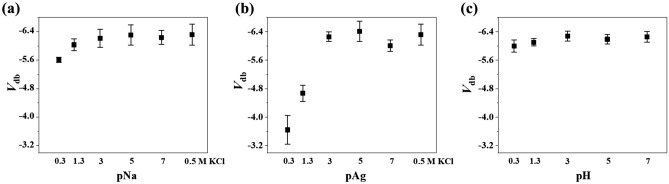
Potential at which dielectric breakdown occurs (*V*_db_) for SiO_2_ as a function of (**a**) sodium ion concentration, (**b**) silver ion concentration, and (**c**) proton concentration.

To investigate cationic effect on DB in EOS system, the *i*–V curves were obtained in the presence of alkali metal ions (Li^+^, Na^+^, K^+^, Cs^+^) and silver ion (Ag^+^). The results from solutions containing NaCl and AgNO_3_ are conspicuous in that *V*_db_ positively shifts for SiO_2_ (Fig. 2a). Plasma-enhanced chemical vapor deposition (PECVD) SiO_2_ also showed a similar *V*_db_ trend (Fig. S1), indicating that it is a feature shared by the SiO_2_ material rather than the method for fabricating thin insulating film. This should be attributed to effects of sodium and silver cations because anionic effect is negligible (Fig. S2). The cationic effect on the breakdown is a function of concentration. Figure 3 shows that both sodium ion, silver ion, and proton have little influence on breakdown of SiO_2_ at concentrations below 1 mM. Furthermore, silver ion causes *V*_db_ to shift to a much greater extent than sodium ion, V_db_ being approximately 2.5 V and 0.7 V, respectively. *V*_db_ shift of proton is 0.25 V and more similar to sodium ion than silver ion. It is increasingly probable that SiO_2_ film undergoes breakdown at lower voltage, i.e. weaker electric field, in the presence of sodium ion, silver ion and proton at concentration higher than 1 mM.

**Figure 4:**
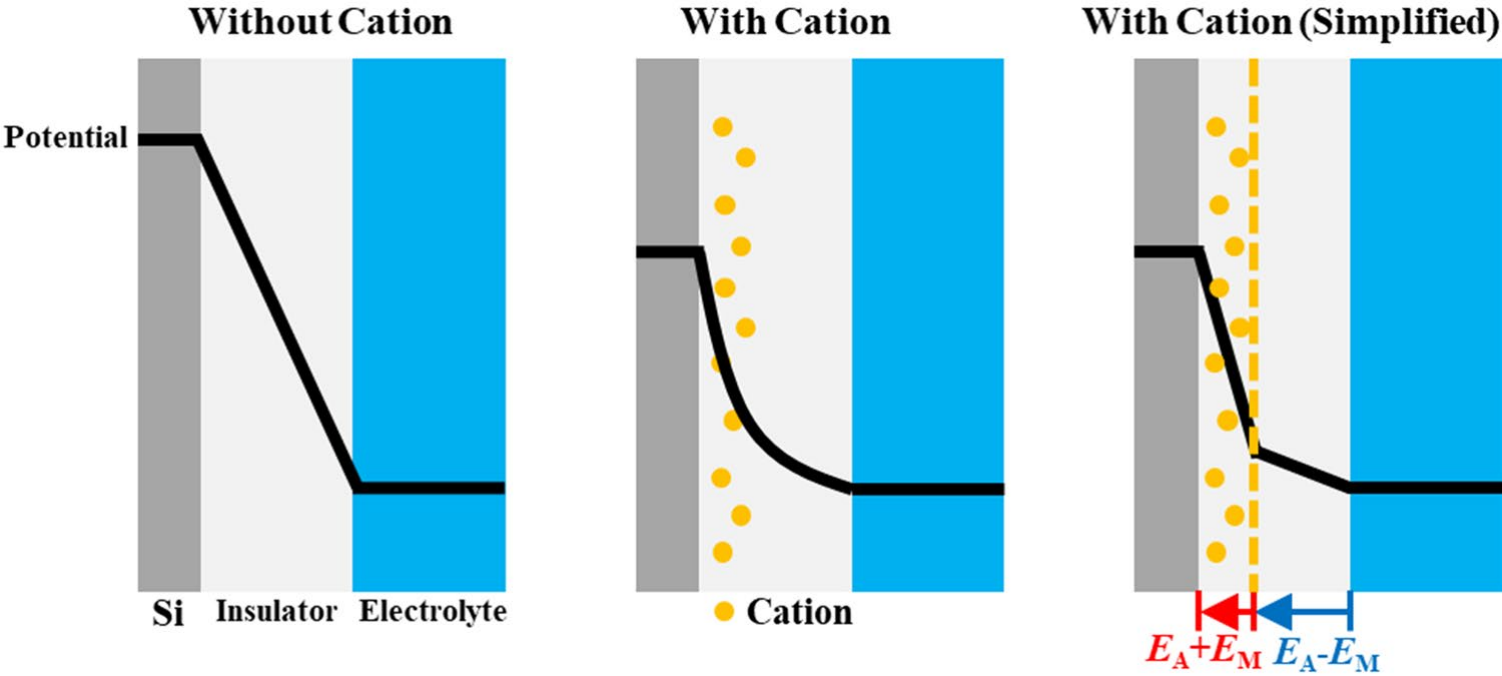
Potential diagrams of Si–SiO_2_–electrolyte systems with or without transferrable cations in electrolyte.

Concerning the influence of sodium and silver ions on DB of SiO_2_, there are a few reports that mobile cations that forcibly impregnated in dielectric films during the fabrication lower *V*_db_ increasingly with effective electric field (Fig. 4)^20,21^. In this system, metal ions are initially absent in the SiO_2_ film and present in the solution. Therefore, the cations should transfer from the aqueous phase to the SiO_2_ layer upon negative electric bias.

Based on the assumption that the transferred sodium and silver ions weaken the dielectric strength by modifying the effective electric field, we estimated the number of sodium and silver ions transferred into SiO_2_ by employing the methodology to analyze the introduction of cations to hydrogenated silicon carbon nitride film^21^. Briefly, we positioned sodium or silver ions on a plane and calculated the effective electric field by summation of the applied field and the field generated by the ions. Potential differences at the interfaces of Si–SiO_2_ and SiO_2_–electrolyte are not considered because the applied voltage is significantly larger. Thus, the magnitude of applied field gradient, *E*_A_, would be *V*_A_/*d* where *V*_A_ is applied voltage between reference electrode and Si and *d* is the oxide thickness. By the Gauss law, electric field created by sodium ion on a plane in the film should have the magnitude of *E*_M_ = *qN*_M_/2ε where *q* is the charge of electron, *N*_M_ is area density of sodium or silver ion, *ε* is the permittivity of free space. Thus, the electric field applied between Si and the plane of sodium or silver ion is *E*_total_ = *E*_A_ + *E*_M_ = *V*_A_/*d* + *qN*_M_/2ε. When *E*_total_ exceeds the dielectric strength of SiO_2_, which is *V*_db_/*d* in the absence of sodium and silver ions, breakdown should occur. Using the measured *V*_db_ in Fig. 2a, we estimated the number of ions per unit area of 10 nm-thick film, 7.67 × 10^15^ m^−2^, one sodium ion per 3000 SiO_2_, or 13 mM. In the same way, the estimated number of silver ions per unit area of 10 nm-thick film is 2.94 × 10^16^ m^−2^, one silver ion per 780 SiO_2_, or 49 mM.

**Figure 5:**
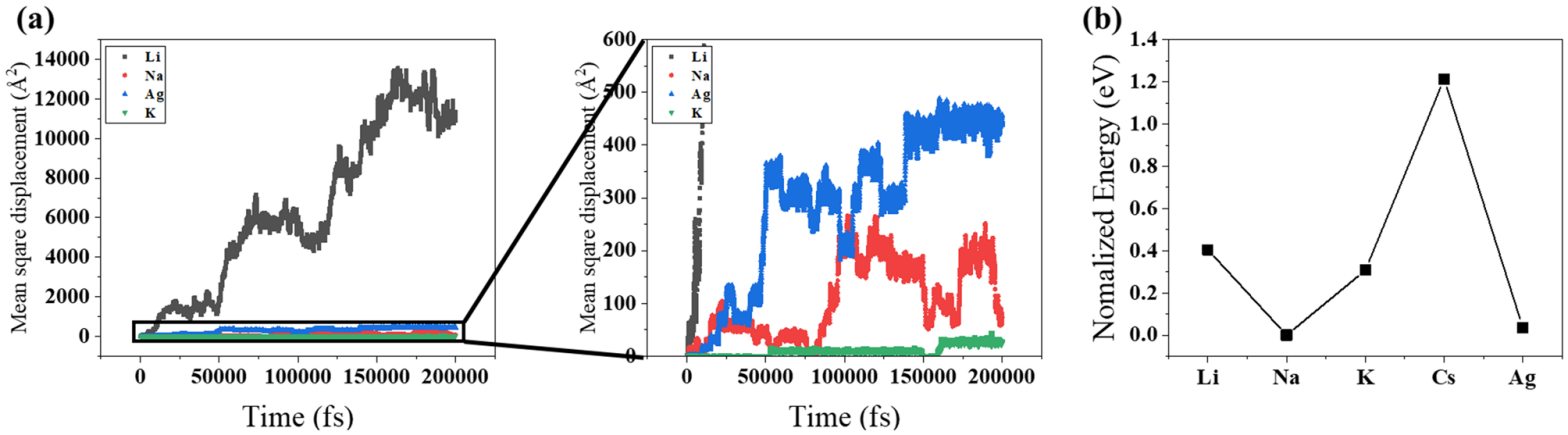
(**a**) Mean square displacement of cations versus time for different metal ions calculated by AIMD simulation. (**b**) Normalized free energy required for cation transport from aqueous to SiO_2_ phase.

As for transportation of cation between two different phases, we can divide its entire process into 3 steps. (1) The solvation shell of the cation in solution phase would be completely or partially removed before entering the oxide phase. (2) In the oxide, the lattice would get distorted or locally reduced to stabilize the local excess charge. (3) At the interface, the adsorption of the cation on the terminal group of the oxide could catalyze the interphase transport process by lowering activation energy. In order to further elucidate the cation selectivity of SiO_2_-aqueous system, we investigated transportability of metal ions in SiO_2_ structure by ab-initio molecular dynamics (AIMD) simulations and theoretical calculations based on density functional theory (DFT) by assessing the relative stability of metal cations. The mean square displacement of each metal ion calculated by AIMD is depicted in Fig. 5a as a function of simulation time. In order to confirm the smooth movement of ions, we monitored it in the fs time scale at 1800 K. The movement of cations is observed inside SiO_2_. Larger metal ions such as potassium ions can barely migrate in SiO_2_ matrix, implying that they are less likely to contribute the breakdown enhancement even if the cations intercalate into the first layer of the matrix. In contrast, the ionic motion of lithium ion is much faster than that of other ions. Drifts of sodium and silver ions are moderately fast. Therefore, we can predict that lithium, sodium and silver ions may affect the breakdown potential once they are transferred from the electrolyte phase. However, the observed *V*_db_ shifts cannot be fully addressed, until the solvation energy of each ions are considered. Figure 5b shows the normalized relative stabilities of metal cations in SiO_2_ compared to water. We normalized the data using the lowest value, which corresponds to that of sodium ion, for comparison. Positive values for lithium, potassium and cesium ions show that they are not likely to reside in the SiO_2_ phase compared with sodium and silver ions. The driving force for the insertion from water to SiO_2_ is the highest for sodium and silver ions. Putting together the AIMD simulations and the DFT energy calculations, we can explain why sodium and silver ions modify *V*_db_ significantly unlike other alkali metal ions. Computational analysis says that the apparent *V*_db_ shift is driven by desolvation energy rather than movement within the SiO_2_ phase. Lithium ion has negligible effect on *V*_db_ because its insertion from aqueous phase to SiO_2_ is energetically unfavorable (Fig. 5b). According to literature, anions can influence the adsorption of alkali metal ions on SiO_2_ surface and considerable adsorption of sodium ion is expected when Cl^−^ coexists^36^. Reportedly, the partially solvated cation can be adsorbed on the dielectric film and such an intermittent state may affect the cation transfer. Small change in *V*_db_ (Fig. S2), however, indicates that this is not the case for this system.

In DFT energy calculation and AIMD simulation, sodium ions and silver ions are almost similar, but as shown in Fig. 3, silver ions have a great effect compared to sodium ions. If the difference between the sodium ion and the silver ion present is that silver ion can be metallization by a weak electric field within the SiO_2_^37^. The metalized silver inside the SiO_2_ becomes a site for current flow and leads to a breakdown soon, but sodium ions and similar protons do not lead directly to breakdown because they exist only as cations inside SiO_2_.

Neither sodium nor silver effect on breakdown potential is observed at Si_3_N_4_ (Fig. 2b), showing that cationic effect is very sensitive to the differences in chemical nature between SiO_2_ and Si_3_N_4_. For HfO_2_, silver ion lowers the *V*_db_ whereas sodium ion has negligible influence. *V*_db_ shift at HfO_2_ is observed only for silver ion to 1.2 V, less than for SiO_2_.

AIMD simulations were also performed on Si_3_N_4_ and HfO_2_ to see transportability of metal ions. AIMD simulations were performed on hexagonal Si_3_N_4_ and monoclinic HfO_2_ where ion movement was shown only in 1D channel. This 1D channel ion pathway could easily be inhibited due to path-blocking defects^38,39^, unlike 3D channel of SiO_2_. Since the actual Si_3_N_4_ used in the experiments was amorphous possessing numerous defect sites, it is hardly expected that ions drifts in Si_3_N_4_ affect the breakdown (Fig. 2b). As for HfO_2_, AIMD simulation shows that all cations do not move, even though strong silver ion induced *V*_db_ lowering was observed in the experiment (Fig. 2c). The difference between the experiment and the simulation could be addressed by the structural variation in the oxides used by each method. The HfO_2_ structure we used for AIMD simulation was crystalline HfO_2,_ free of structural defects. In the experiment amorphous HfO_2_ was used. Unlike the crystalline oxide, amorphous has many different kinds of defects that can allow flow of charge or mass. Silver influence was observed at HfO_2_ as well as at SiO_2_ while not at Si_3_N_4_, suggesting the role of defects in the ionic conduction in the oxide layers. For HfO_2_, oxygen vacancy is considered a main cause of conduction^40^ which could be intrinsically present after the fabrication or possibly be generated under strong electric field. The amorphous HfO_2_ film used in the breakdown experiments has a high population of oxygen vacancy due to the stoichiometric imbalance. We assume that these oxygen vacancies may provide a pathway for silver ions movement. In order to find out the relationship between the oxygen vacancy and the conduction of silver ions, oxygen vacancy adjustment was performed. The oxygen vacancies in HfO_2_ can be reduced through UV/ozone plasma treatment^41^. The decrease in the population of oxygen vacancies after the treatment was confirmed via X-ray photoelectron spectrum (Fig. S3a). *V*_db_ of HfO_2_ was shifted negatively according to UV/ozone plasma treatment by 0.35 V (Fig. S3b), which indicates silver effect on breakdown potential of HfO_2_ was weakened with the decrease in oxygen vacancies. The dielectric strength was preserved after the plasma treatment according to the unchanged *V*_db_ in potassium based electrolyte. Given these experimental results, the presence of oxygen vacancies supposedly aids silver ions movement into HfO_2_.

## Conclusions

In this study, we examined the effect of alkali metal and silver ions on the dielectric layer of Si-insulator-electrolyte (EOS) system under the condition of negative electrical bias. Like metal–insulator-electrolyte (MOS) system, the breakdown of insulator in EOS system is observed and its potential, *V*_db_, depends on the type of dielectric material. *V*_db_ of the Si_3_N_4_, remains constant regardless of the monovalent metal ions investigated in this work. On the other hand, the most widely used insulator, SiO_2_, is vulnerable to sodium and silver ions when the solution concentration is higher than 1 mM. HfO_2_ film is resistant to sodium ion but significantly influenced by silver ion. The oxygen vacancies of HfO_2_ may interact with silver ion. The discriminative influence of cations upon electrical bias in the EOS system was examined in two aspects, molecular dynamics and desolvation of the cations. Lithium, sodium and silver ions clearly show that the breakdown of the dielectric films, *V*_db_, is driven predominantly by desolvation of ions, which is in contrast to MOS systems.

## Materials and methods

### Materials

Phosphate buffer solution (PBS) was made using phosphoric acid (Daejung, Korea), potassium phosphate monobasic and potassium phosphate dibasic (99.9%) from Sigma-Aldrich. Highly doped n-type silicon wafer (arsenic-doped, < 100 > -oriented) with a resistivity as low as 0.005 Ω cm was obtained from STC (Japan). AZ4620 photoresist was purchased from Merck (USA).

### Preparation of thermal oxidized Si/SiO_2_, Si/Si_3_N_4_ and Si/HfO_2_

The 10-nm thick thermal SiO_2_ film was prepared by dry oxidation of Si wafer in oxygen environment at 950 °C. Briefly, Si wafer was cleaned with a mixture of H_2_SO_4_ and H_2_O_2_ before stripping the native oxide by dipping in HF. A 20-nm-thick thermal SiO_2_ layer was produced at 850 °C in a furnace with dry O_2_ blowing followed by HF wet etching. Repetitive cleaning was followed by dry O_2_ blowing in a furnace at 950 °C yielding 10-nm-thick thermal SiO_2_ layers. 10-nm-thick Si_3_N_4_ was obtained by low pressure chemical vapor deposition (LPCVD) after standard wafer cleaning process. 10-nm HfO_2_ layers were deposited by atomic layer deposition (ALD) on same cleaned Si wafer. Tetrakis(ethylmethylamido)hafnium was used as the metal precursor, and deionized H_2_O as the oxygen source. The temperature in the ALD reaction chamber was 200 °C.

### Protective layer coatings on Si/SiO_2_, Si/Si_3_N_4_ and Si/HfO_2_

For reproducible experiments we had to define the exposed SiO_2_ area and reduce unwanted pinholes in the oxide film. Successive coatings of alumina, photoresist and photolithography were performed as follows. First of all, 100 nm-thick alumina film was deposited on the whole wafer of Si/SiO_2_ by a SNTEK sputter. AZ4620 photoresist was spin coated on the wafer at 6000 rpm for 30 s and post baking process was conducted at 110 °C for 1.5 min. Then we aligned the wafer under a pattern-designed chromium mask (50 × 50 μm^2^), and exposed it to a UV lamp. Developing was performed by immersing the wafer into AZ300MIF developer (AZ electronic materials) for 2 min. After judging whether the wafer was well developed by optical microscope, a hard bake was performed at 150 °C for 15 min. Etching of alumina was conducted in 50 °C, 85% phosphoric acid for 6 min.

### Electrochemical characterization

To minimize mechanical stress, we used the whole wafer without a dicing process for all electrochemical experiments^17^. For electrical contact with Si/SiO_2_, Si/Si_3_N_4_ and Si/HfO_2_, the insulating layer on the back of silicon wafer was removed by scratching an approximately ~ 1 cm^2^ area with a diamond point pen and casting a droplet of 48% hydrofluoric acid solution^17^. This area was covered by gallium-indium eutectic (≥ 99.99% trace metals basis from Sigma-Aldrich) then covered by ~ 10-cm-long conductive adhesive tape^17^. The tape was connected to the working electrode cable of the electrochemical analyzer (CHI660, CH Instrument, US)^17^. 3 μL of 0.5 M aqueous electrolyte was dropped on the exposed SiO_2_ area^17^. To complete the electrochemical cell Pt wire and Ag/AgCl reference electrode (3 M KCl) with a double junction filled with 1 M KNO_3_ were employed as the counter and reference electrodes, respectively. All potentials in this paper are referenced to Ag/AgCl reference electrode (3 M KCl)^17^. Linear sweep voltammetry (LSV) was carried out to see characteristic dielectric breakdown behavior of Si/SiO_2_, Si/Si_3_N_4_ and Si/HfO_2_. LSV initial potential for SiO_2_ and Si_3_N_4_ was—3 V, and for HfO_2_ was 0 V. The scan rate was 50 mV/s. Potential at which dielectric breakdown occurred (*V*_db_) was determined as the most positive potential with 100 nA during LSV.

### UV/ozone plasma treatment

HfO_2_ oxidation for eliminating oxygen vacancy was conducted with UV/ozone plasma treatment (AC6, Ahtech LTS, Korea) for 30 min while the distance between the UV lamp and the substrates was maintained at 45 mm.

### Theoretical calculation

Total energy calculations were performed by using Vienna ab initio Simulation Package (VASP) based on density functional theory^42^. We applied the generalized gradient approximation with the Perdew–Burke–Ernzerhof (PBE) function to treat the exchange–correlation energy^43^. DFT energy calculations were conducted spin-polarized. A kinetic energy cutoff of 600 eV and a *Γ*-point-only *k*-point grid were used, which conditions showed negligible difference with the calculation using a kinetic energy cutoff of 800 eV and a 2 × 2 × 2 k-point grid based on Monkhorst–Pack scheme^44^. To describe the SiO_2_ system, we adopted a 2 × 2 × 2 supercell of *α*-cristobalite structure (space group: P4_1_2_1_2) and let it fully relax until the residual force was less than 0.02 eV/Å. We estimated the relative stability of various alkali metal ions in between two mediums. One is in the solution, and the other is in interstitial sites in SiO_2_. The relative stability, $\Delta E$, can be expressed as:

$$ \Delta E\left( x \right) = E_{{x\; in\;SiO_{2} }} \left( x \right) - E_{{SiO_{2} }} - \left\{ {E_{x\;in\;vacuum} \left( x \right) + E_{solvation} \left( x \right)} \right\} $$

where $x$ is alkali metal ion such as Li^+^, Na^+^, K^+^, Rb^+^, Cs^+^ and Ag^+^. To determine the most stable interstitial sites in SiO_2_, we considered seven different interstitial sites generated from Voronoi analysis^45^. Subsequently, the lowest-DFT-energy structure was selected to estimate $E_{{x\;in\;SiO_{2} }} \left( x \right)$ that contains one alkali metal ion. Only the internal atomic positions were allowed relax while the lattice constants were fixed to those of the fully-relaxed SiO_2_ structure when we calculate $E_{{x\;in\;SiO_{2} }} \left( x \right)$. $E_{{SiO_{2} }}$ is the DFT energy of 2 × 2 × 2 supercell of *α*-cristobalite structure. Energy of single alkali metal ion in the vacuum, $E_{x \;in\;vacuum} \left( x \right)$, was obtained from a 20 × 20 × 20 Å^3^ cubic cell with one ion in the box. The energy of the single ion was corrected by using solvation energy, $E_{solvation} \left( x \right)$, to estimate the energy of ion in solvated states (Table S1). Ab initio molecular dynamics simulations were carried out by employing VASP based on Verlet algorithm^46^. The same structures which were used for calculating $E_{{x \;in\;SiO_{2} }} \left( x \right)$ were adopted for AIMD simulations except for the Cs^+^-inserted SiO_2_ case where the electronic energy could not be converged in AIMD calculation conditions. We also did AIMD simulations on hexagonal Si_3_N_4_ (space group: P6_3_/m) and monoclinic HfO_2_ (space group: P2_1_/c) using a *Γ*-point-only *k*-point grid and a cutoff energy of 400 eV. The simulations were performed for 200 ps after an equilibrium step of 10 ps in the canonical (NVT) ensemble with a Nosé–Hoover thermostat^47,48^. All AIMD simulations were conducted at an elevated temperature of 1800 K to facilitate the ionic motions.

## Supplementary information

Supplementary Information 1.
