# Supplementary material for: Aqueous ionic effect on electrochemical breakdown of Si-dielectric–electrolyte interface
Source: Sci Rep. 2020 Oct 8;10:16795. doi: 10.1038/s41598-020-73880-w (PMC7544892; doi:10.1038/s41598-020-73880-w)
Supplement: Supplementary file 1 — Supplementary Information 1. [file 41598_2020_73880_MOESM1_ESM.pdf]

Supplementary Information

Aqueous Ionic Effect on Electrochemical Breakdown of Si-Dielectric-Electrolyte Interface

Jeongse Yun[1,+], Jae Gyeong Lee[1,+], Kyungbae Oh[2], Kisuk Kang[2,3], and Taek Dong Chung[1,*]

[1]Department of Chemistry, Seoul National University, Seoul, 08826, Republic of Korea

[2]Department of Materials Science and Engineering, Research Institute for Advanced Materials (RIAM), Seoul National University, 1 Gwanak-ro, Gwanak-gu, Seoul 08826, Republic of Korea

[3]Institute of Engineering Research, College of Engineering, Seoul National University, 1 Gwanak-ro, Gwanak-gu, Seoul 08826, Republic of Korea

[*]corresponding. (tdchung@snu.ac.kr)

[+]these authors contributed equally to this work

Ion	Li^+	Na^+	K^+	Cs^+	Ag^+
$-\Delta H$/kJ mol^{-1}	531	416	334	283	483

Table S1. Standard Molar Enthalpies of Hydration of Ions.[1]

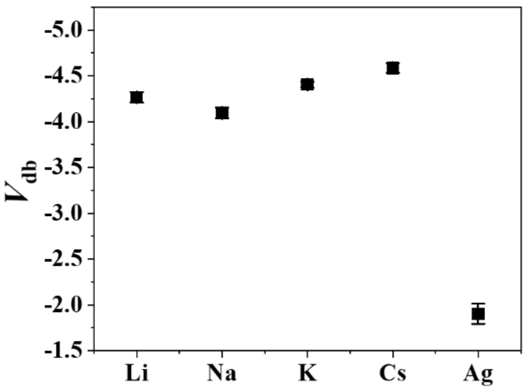

Figure S1. V_{db} of Si-PECVD SiO_2 (10 nm)-aqueous electrolyte in electrolytes containing different metal cations.

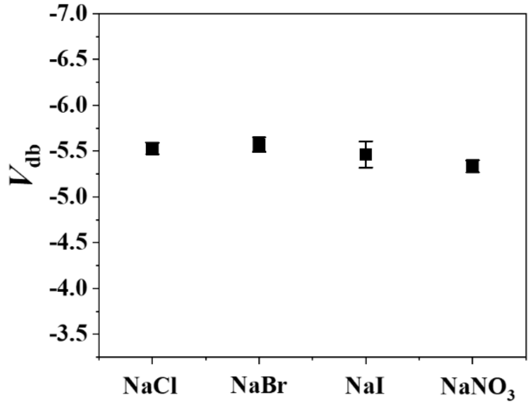

Figure S2. V_{db} of Si-SiO_2 (10 nm)-aqueous electrolyte containing different sodium based electrolytes.

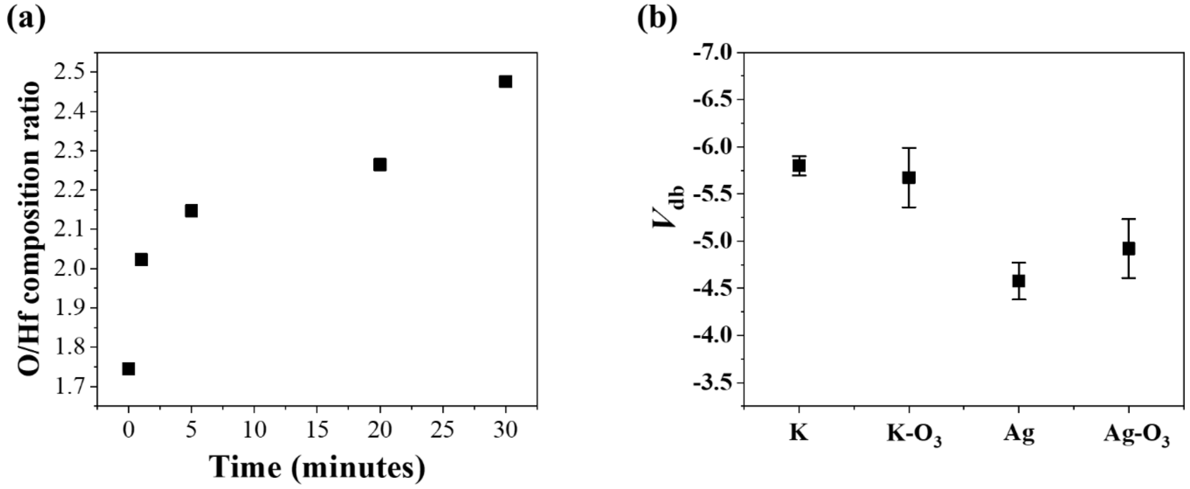

Figure S3. (a) Atomic ratio of oxygen to hafnium for HfO_2 at different UV/ozone plasma treatment times. (b) V_{db} of HfO_2 depending on absence or presence of 30 min UV/ozone plasma treatment.

Reference

1. Yizhak Marcus. *Ions in solution and their Solvation*. (John Wiley & Sons, Inc., Hoboken, New Jersey, 2016).
